# 2,3,4,6-Tetra-*O*-acetyl-β-d-galacto­pyrano­syl butyrate

**DOI:** 10.1107/S1600536811055814

**Published:** 2012-01-11

**Authors:** Yan-Li Cui, Ming-Han Xu, Jian-Wei Mao, Yong-Ping Yu

**Affiliations:** a Department of Chemistry, Zhejiang University, People’s Republic of China; b Department of Biological and Chemical Engineering, Zhejiang University of Science and Technology, People’s Republic of China; c College of Pharmaceutical Sciences, Zhejiang University, People’s Republic of China

## Abstract

The title compound, C_18_H_26_O_11_, was synthesized by a condensation reaction of 2,3,4,6-tetra-*O*-acetyl-α-d-galactopyranosyl bromide and butyric acid. The acet­oxy­methyl and butyrate groups are located on the same side of the pyran ring, showing the β configuration for the d-glycosyl ester; the butyl group adopts an extend conformation, the C—C—C—C torsion angle being 179.1 (7)°. In the crystal, the mol­ecules are linked by weak C—H⋯O hydrogen bonds.

## Related literature

For the total synthesis of glycosyl esters, see: Li *et al.* (1992 ▶); Smith *et al.* (1986 ▶). For the anti-tumor activities of glycosyl esters, see: Feldman *et al.* (2000 ▶). For related structures, see: Sambaiah *et al.* (2001 ▶); Parkanyi *et al.* (1987 ▶); Roslund *et al.* (2004 ▶); Liu *et al.* (2009 ▶); Kumar *et al.* (2005) ▶. For the synthesis, see: Loganathan & Trivedi (1987 ▶).
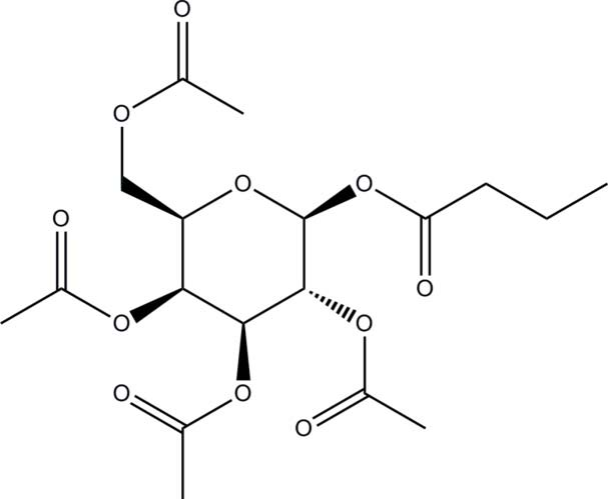


## Experimental

### 

#### Crystal data

C_18_H_26_O_11_
*M* *_r_* = 418.39
Monoclinic, 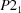

*a* = 9.2079 (9) Å
*b* = 8.5034 (5) Å
*c* = 14.3199 (12) Å
β = 100.804 (9)°
*V* = 1101.35 (16) Å^3^
*Z* = 2
Mo *K*α radiation
μ = 0.11 mm^−1^
*T* = 294 K
0.38 × 0.32 × 0.25 mm

#### Data collection

Oxford Diffraction Xcalibur Atlas Gemini Ultra diffractometer
6393 measured reflections
2160 independent reflections
1582 reflections with *I* > 2σ(*I*)
*R*_int_ = 0.028

#### Refinement

*R*[*F* ^2^ > 2σ(*F* ^2^)] = 0.048
*wR*(*F* ^2^) = 0.147
*S* = 1.03
2160 reflections
268 parameters
1 restraint
H-atom parameters constrained
Δρ_max_ = 0.35 e Å^−3^
Δρ_min_ = −0.21 e Å^−3^

### 

Data collection: *CrysAlis PRO* (Oxford Diffraction, 2007 ▶); cell refinement: *CrysAlis PRO*; data reduction: *CrysAlis RED* (Oxford Diffraction, 2007 ▶); program(s) used to solve structure: *SHELXTL* (Sheldrick, 2008 ▶); program(s) used to refine structure: *SHELXTL*; molecular graphics: *SHELXTL*; software used to prepare material for publication: *SHELXTL*.

## Supplementary Material

Crystal structure: contains datablock(s) I, global. DOI: 10.1107/S1600536811055814/xu5405sup1.cif

Structure factors: contains datablock(s) I. DOI: 10.1107/S1600536811055814/xu5405Isup2.hkl

Supplementary material file. DOI: 10.1107/S1600536811055814/xu5405Isup3.cml

Additional supplementary materials:  crystallographic information; 3D view; checkCIF report

## Figures and Tables

**Table 1:** Hydrogen-bond geometry (Å, °)

*D*—H⋯*A*	*D*—H	H⋯*A*	*D*⋯*A*	*D*—H⋯*A*
C3—H3⋯O11^i^	0.98	2.47	3.362 (5)	152
C5—H5⋯O11^i^	0.98	2.57	3.443 (6)	149
C11—H11*B*⋯O5^ii^	0.96	2.49	3.293 (7)	141
C16—H16*C*⋯O9^iii^	0.96	2.60	3.441 (7)	147

Symmetry codes: (i) 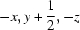; (ii) 
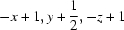
; (iii) 
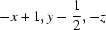
.

**Fig. 1.**
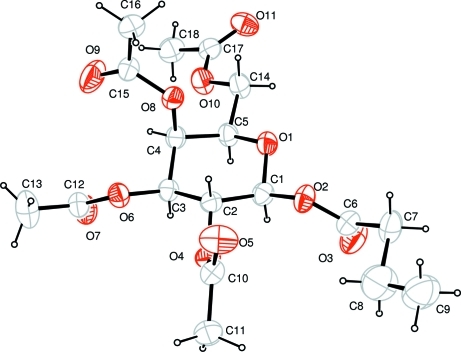
The molecular structure of (I) with 30% probability displacement ellipsoids.

**Fig. 2.**
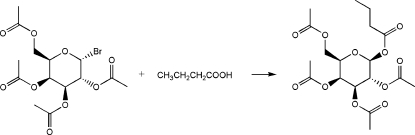
Reaction scheme.

## supplementary crystallographic information

### Comment

Carbohydrates provide excellent platforms upon which to explore unique features for the drug-discovery process. Numerous natural glycosyl esters such as phyllanthostatin family (Li *et al.*, 1992) and dimeric ellagitannin coriariin A have been total synthetized (Smith *et al.*, 1986). Some of them were proved to possess anti-tumor activities (Feldman *et al.*, 2000). Also the glycosyl esters have long drawn attention as potential glycosyl donors. Several crystal structures of carbohydrate derivatives were reported (Sambaiah *et al.*, 2001; Parkanyi *et al.*, 1987; Roslund *et al.*, 2004; Liu *et al.*, 2009; Kumar *et al.*, 2005). Recently we have synthetized the title compound and report its crystal structure herein.

The molecular structure of the title compound is shown in Fig. 1. In the molecule, the acetoxymethyl and butyrate groups are located on the same side of the pyran ring, showing the β-configuration for the D-glycosyl ester; the butyl group adopts an extend conformation, the C6–C7–C8–C9 torsion angle being 179.1 (7)°. The molecules are linked by weak C—H···O hydrogen bonding in the crystal.

### Experimental

A solution of butyric acid (48.8 µl, 0.53 mmol), tetrabutylammonium iodide (26.7 mg, 0.07 mmol) and 5% aqueous sodium hydroxide (2 ml, 17.1 mg, 1.35 mmol) in dichloromethane (2 ml) was vigorously stirred at room temperature, then 2,3,4,6-tetra-*O*-acetyl-α-D-galactopyranosyl bromide (145.1 mg, 0.35 mmol) was added. The mixture was stirred for 30 h, the two phases (dichloromethane phase and water phase) were then separated. The organic layer was washed with a sodium hydroxide aqueous solution (5%) and water for several times, dried over sodium sulfate, filtered and concentrated. The residue was purified by silica gel chromatography (petroether/EtOAc = 2:1) to afford the title compound (Fig. 2). Single crystals suitable for X-ray data collection were obtained by slow evaporation from an ether solution (Loganathan *et al.*, 1987).

### Refinement

Methyl H atoms were placed in calculated position with C—H = 0.96 Å and torsion angle was refined from electron density with *U*_iso_(H) = 1.5*U*_eq_(C). Other H atoms were placed in calculated positions with C—H = 0.97–0.98 Å, and included in the final cycles of refinement in riding model with *U*_iso_(H) = 1.2*U*_eq_(C). As no significant anomalous scatterings, Friedel pairs were merged. The enantiomer has been assigned by reference to the unchanging chiral C5 atom in the synthetic procedure.

### Crystal data

**Table:**

C_18_H_26_O_11_	*F*(000) = 444
*M**_r_* = 418.39	*D*_x_ = 1.262 Mg m^−^^3^
Monoclinic, *P*2_1_	Mo *K*α radiation, λ = 0.71073 Å
Hall symbol: P 2yb	Cell parameters from 3799 reflections
*a* = 9.2079 (9) Å	θ = 3.2–26.4°
*b* = 8.5034 (5) Å	µ = 0.11 mm^−^^1^
*c* = 14.3199 (12) Å	*T* = 294 K
β = 100.804 (9)°	Block, colorless
*V* = 1101.35 (16) Å^3^	0.38 × 0.32 × 0.25 mm
*Z* = 2	

### Data collection

**Table:**

Oxford Diffraction Xcalibur Atlas Gemini ultra diffractometer	1582 reflections with *I* > 2σ(*I*)
Radiation source: fine-focus sealed tube	*R*_int_ = 0.028
graphite	θ_max_ = 25.4°, θ_min_ = 2.9°
Detector resolution: 10.3592 pixels mm^-1^	*h* = −11→8
ω scans	*k* = −10→9
6393 measured reflections	*l* = −16→17
2160 independent reflections	

### Refinement

**Table:**

Refinement on *F*^2^	Secondary atom site location: difference Fourier map
Least-squares matrix: full	Hydrogen site location: inferred from neighbouring sites
*R*[*F*^2^ > 2σ(*F*^2^)] = 0.048	H-atom parameters constrained
*wR*(*F*^2^) = 0.147	*w* = 1/[σ^2^(*F*_o_^2^) + (0.0855*P*)^2^ + 0.0985*P*] where *P* = (*F*_o_^2^ + 2*F*_c_^2^)/3
*S* = 1.03	(Δ/σ)_max_ = 0.002
2160 reflections	Δρ_max_ = 0.35 e Å^−^^3^
268 parameters	Δρ_min_ = −0.21 e Å^−^^3^
1 restraint	Extinction correction: *SHELXTL* (Sheldrick, 2008), Fc^*^=kFc[1+0.001xFc^2^λ^3^/sin(2θ)]^-1/4^
Primary atom site location: structure-invariant direct methods	Extinction coefficient: 0.022 (6)

### Special details

**Table:**

Geometry. All e.s.d.'s (except the e.s.d. in the dihedral angle between two l.s. planes) are estimated using the full covariance matrix. The cell e.s.d.'s are taken into account individually in the estimation of e.s.d.'s in distances, angles and torsion angles; correlations between e.s.d.'s in cell parameters are only used when they are defined by crystal symmetry. An approximate (isotropic) treatment of cell e.s.d.'s is used for estimating e.s.d.'s involving l.s. planes.
Refinement. Refinement of *F*^2^ against ALL reflections. The weighted *R*-factor *wR* and goodness of fit *S* are based on *F*^2^, conventional *R*-factors *R* are based on *F*, with *F* set to zero for negative *F*^2^. The threshold expression of *F*^2^ > σ(*F*^2^) is used only for calculating *R*-factors(gt) *etc*. and is not relevant to the choice of reflections for refinement. *R*-factors based on *F*^2^ are statistically about twice as large as those based on *F*, and *R*- factors based on ALL data will be even larger.

### Fractional atomic coordinates and isotropic or equivalent isotropic displacement parameters (Å^2^)

**Table:**

	*x*	*y*	*z*	*U*_iso_*/*U*_eq_	
O1	0.1960 (3)	0.9067 (3)	0.2033 (2)	0.0627 (8)	
O2	0.2171 (3)	0.8828 (4)	0.3617 (2)	0.0669 (8)	
O3	−0.0223 (5)	0.8472 (6)	0.3587 (3)	0.1095 (14)	
O4	0.2776 (3)	1.2097 (3)	0.3854 (2)	0.0599 (7)	
O5	0.4753 (4)	1.1092 (5)	0.4784 (2)	0.0989 (13)	
O6	0.4015 (3)	1.3413 (3)	0.23619 (19)	0.0606 (7)	
O7	0.2592 (4)	1.5007 (4)	0.1320 (3)	0.0843 (10)	
O8	0.4303 (3)	1.0527 (3)	0.13984 (19)	0.0607 (7)	
O9	0.5101 (5)	1.2274 (5)	0.0433 (3)	0.1129 (15)	
O10	0.1343 (4)	1.0060 (4)	−0.0433 (2)	0.0778 (9)	
O11	0.0730 (4)	0.8056 (4)	−0.1411 (2)	0.0833 (10)	
C1	0.1872 (5)	0.9923 (5)	0.2865 (3)	0.0579 (10)	
H1	0.0883	1.0375	0.2824	0.069*	
C2	0.3038 (4)	1.1195 (5)	0.3053 (3)	0.0516 (9)	
H2	0.4030	1.0731	0.3185	0.062*	
C3	0.2868 (4)	1.2263 (4)	0.2199 (3)	0.0524 (10)	
H3	0.1912	1.2800	0.2127	0.063*	
C4	0.2904 (4)	1.1312 (5)	0.1311 (3)	0.0553 (10)	
H4	0.2741	1.1999	0.0751	0.066*	
C5	0.1711 (5)	1.0062 (5)	0.1214 (3)	0.0592 (10)	
H5	0.0749	1.0579	0.1171	0.071*	
C6	0.1034 (6)	0.8206 (6)	0.3940 (3)	0.0691 (12)	
C7	0.1506 (7)	0.7109 (7)	0.4739 (3)	0.0898 (16)	
H7A	0.2396	0.6558	0.4658	0.108*	
H7B	0.0737	0.6338	0.4764	0.108*	
C8	0.1820 (10)	0.8143 (11)	0.5717 (4)	0.139 (3)	
H8A	0.2591	0.8907	0.5683	0.166*	
H8B	0.0931	0.8714	0.5779	0.166*	
C9	0.2258 (9)	0.7182 (11)	0.6526 (4)	0.135 (3)	
H9A	0.3178	0.6677	0.6490	0.202*	
H9B	0.1515	0.6398	0.6548	0.202*	
H9C	0.2378	0.7817	0.7090	0.202*	
C10	0.3699 (5)	1.1938 (6)	0.4686 (3)	0.0640 (11)	
C11	0.3271 (7)	1.2933 (7)	0.5437 (3)	0.0857 (15)	
H11A	0.2395	1.3516	0.5177	0.129*	
H11B	0.4059	1.3650	0.5674	0.129*	
H11C	0.3080	1.2280	0.5947	0.129*	
C12	0.3736 (6)	1.4773 (5)	0.1855 (4)	0.0698 (12)	
C13	0.4984 (7)	1.5887 (8)	0.2062 (5)	0.114 (2)	
H13A	0.5798	1.5396	0.2476	0.171*	
H13B	0.4683	1.6807	0.2364	0.171*	
H13C	0.5283	1.6182	0.1479	0.171*	
C14	0.1652 (6)	0.9010 (6)	0.0361 (3)	0.0709 (12)	
H14A	0.2590	0.8479	0.0381	0.085*	
H14B	0.0878	0.8229	0.0331	0.085*	
C15	0.5298 (5)	1.1116 (6)	0.0906 (3)	0.0697 (12)	
C16	0.6667 (5)	1.0135 (6)	0.1061 (4)	0.0839 (15)	
H16A	0.7105	1.0135	0.1724	0.126*	
H16B	0.7356	1.0562	0.0701	0.126*	
H16C	0.6420	0.9077	0.0856	0.126*	
C17	0.0882 (5)	0.9440 (6)	−0.1293 (3)	0.0653 (12)	
C18	0.0590 (7)	1.0649 (8)	−0.2023 (3)	0.0884 (15)	
H18A	0.1422	1.1347	−0.1960	0.133*	
H18B	−0.0274	1.1233	−0.1950	0.133*	
H18C	0.0429	1.0165	−0.2639	0.133*	

### Atomic displacement parameters (Å^2^)

**Table:**

	*U*^11^	*U*^22^	*U*^33^	*U*^12^	*U*^13^	*U*^23^
O1	0.0752 (19)	0.0537 (15)	0.0613 (17)	−0.0060 (14)	0.0180 (14)	0.0060 (14)
O2	0.0644 (18)	0.0700 (19)	0.0679 (18)	−0.0023 (15)	0.0161 (14)	0.0242 (16)
O3	0.083 (3)	0.128 (4)	0.126 (3)	−0.001 (2)	0.042 (2)	0.034 (3)
O4	0.0617 (17)	0.0651 (17)	0.0542 (15)	0.0114 (14)	0.0146 (12)	0.0022 (14)
O5	0.084 (2)	0.123 (3)	0.082 (2)	0.028 (3)	−0.0029 (19)	−0.015 (2)
O6	0.0665 (18)	0.0549 (16)	0.0591 (15)	−0.0089 (13)	0.0083 (13)	0.0000 (14)
O7	0.088 (2)	0.068 (2)	0.093 (2)	−0.0005 (18)	0.008 (2)	0.0190 (19)
O8	0.0660 (18)	0.0577 (15)	0.0618 (16)	0.0012 (13)	0.0209 (13)	0.0072 (14)
O9	0.110 (3)	0.112 (3)	0.131 (3)	0.010 (3)	0.061 (3)	0.055 (3)
O10	0.106 (2)	0.0663 (19)	0.0575 (17)	−0.0130 (18)	0.0052 (16)	0.0012 (16)
O11	0.086 (2)	0.080 (2)	0.079 (2)	0.0041 (19)	0.0024 (17)	−0.0216 (19)
C1	0.056 (2)	0.061 (2)	0.056 (2)	0.002 (2)	0.0102 (18)	0.009 (2)
C2	0.053 (2)	0.053 (2)	0.049 (2)	0.0056 (18)	0.0099 (16)	0.0022 (18)
C3	0.050 (2)	0.052 (2)	0.054 (2)	0.0009 (18)	0.0065 (17)	0.0065 (18)
C4	0.060 (2)	0.053 (2)	0.052 (2)	0.0007 (19)	0.0090 (17)	0.0099 (19)
C5	0.070 (3)	0.053 (2)	0.054 (2)	−0.006 (2)	0.0084 (19)	0.0048 (19)
C6	0.075 (3)	0.073 (3)	0.063 (3)	−0.003 (3)	0.023 (2)	0.005 (2)
C7	0.103 (4)	0.091 (3)	0.076 (3)	−0.020 (3)	0.018 (3)	0.024 (3)
C8	0.197 (9)	0.135 (6)	0.081 (4)	0.022 (6)	0.016 (5)	0.021 (4)
C9	0.153 (7)	0.160 (8)	0.095 (4)	−0.013 (6)	0.033 (4)	−0.010 (5)
C10	0.063 (3)	0.070 (3)	0.062 (3)	−0.003 (2)	0.018 (2)	0.004 (2)
C11	0.114 (4)	0.087 (3)	0.061 (3)	−0.007 (3)	0.028 (3)	−0.007 (3)
C12	0.082 (3)	0.056 (3)	0.075 (3)	−0.010 (2)	0.023 (3)	0.002 (2)
C13	0.125 (5)	0.087 (4)	0.122 (5)	−0.041 (4)	0.005 (4)	0.014 (4)
C14	0.089 (3)	0.057 (2)	0.064 (3)	−0.008 (2)	0.007 (2)	0.004 (2)
C15	0.078 (3)	0.065 (3)	0.073 (3)	−0.014 (2)	0.030 (2)	−0.003 (3)
C16	0.075 (3)	0.080 (3)	0.106 (4)	0.000 (3)	0.040 (3)	−0.010 (3)
C17	0.059 (3)	0.076 (3)	0.061 (3)	−0.004 (2)	0.012 (2)	−0.006 (2)
C18	0.097 (4)	0.101 (4)	0.066 (3)	0.002 (3)	0.012 (3)	0.005 (3)

### Geometric parameters (Å, °)

**Table:**

O1—C1	1.412 (5)	C7—C8	1.634 (9)
O1—C5	1.429 (5)	C7—H7A	0.9700
O2—C6	1.331 (5)	C7—H7B	0.9700
O2—C1	1.412 (5)	C8—C9	1.413 (9)
O3—C6	1.195 (6)	C8—H8A	0.9700
O4—C10	1.334 (5)	C8—H8B	0.9700
O4—C2	1.437 (5)	C9—H9A	0.9600
O5—C10	1.195 (6)	C9—H9B	0.9600
O6—C12	1.363 (5)	C9—H9C	0.9600
O6—C3	1.426 (5)	C10—C11	1.478 (7)
O7—C12	1.197 (6)	C11—H11A	0.9600
O8—C15	1.353 (5)	C11—H11B	0.9600
O8—C4	1.435 (5)	C11—H11C	0.9600
O9—C15	1.190 (6)	C12—C13	1.476 (7)
O10—C17	1.334 (5)	C13—H13A	0.9600
O10—C14	1.432 (5)	C13—H13B	0.9600
O11—C17	1.193 (6)	C13—H13C	0.9600
C1—C2	1.513 (6)	C14—H14A	0.9700
C1—H1	0.9800	C14—H14B	0.9700
C2—C3	1.507 (5)	C15—C16	1.492 (7)
C2—H2	0.9800	C16—H16A	0.9600
C3—C4	1.513 (6)	C16—H16B	0.9600
C3—H3	0.9800	C16—H16C	0.9600
C4—C5	1.516 (6)	C17—C18	1.454 (7)
C4—H4	0.9800	C18—H18A	0.9600
C5—C14	1.506 (6)	C18—H18B	0.9600
C5—H5	0.9800	C18—H18C	0.9600
C6—C7	1.477 (7)		
			
C1—O1—C5	111.2 (3)	H8A—C8—H8B	108.0
C6—O2—C1	118.3 (3)	C8—C9—H9A	109.5
C10—O4—C2	119.0 (3)	C8—C9—H9B	109.5
C12—O6—C3	115.7 (3)	H9A—C9—H9B	109.5
C15—O8—C4	117.8 (3)	C8—C9—H9C	109.5
C17—O10—C14	118.0 (4)	H9A—C9—H9C	109.5
O2—C1—O1	105.7 (3)	H9B—C9—H9C	109.5
O2—C1—C2	107.8 (3)	O5—C10—O4	122.2 (4)
O1—C1—C2	111.6 (3)	O5—C10—C11	125.5 (4)
O2—C1—H1	110.6	O4—C10—C11	112.3 (4)
O1—C1—H1	110.6	C10—C11—H11A	109.5
C2—C1—H1	110.6	C10—C11—H11B	109.5
O4—C2—C3	108.6 (3)	H11A—C11—H11B	109.5
O4—C2—C1	107.7 (3)	C10—C11—H11C	109.5
C3—C2—C1	108.9 (3)	H11A—C11—H11C	109.5
O4—C2—H2	110.5	H11B—C11—H11C	109.5
C3—C2—H2	110.5	O7—C12—O6	122.5 (4)
C1—C2—H2	110.5	O7—C12—C13	125.5 (5)
O6—C3—C2	108.6 (3)	O6—C12—C13	112.0 (4)
O6—C3—C4	111.8 (3)	C12—C13—H13A	109.5
C2—C3—C4	110.2 (3)	C12—C13—H13B	109.5
O6—C3—H3	108.7	H13A—C13—H13B	109.5
C2—C3—H3	108.7	C12—C13—H13C	109.5
C4—C3—H3	108.7	H13A—C13—H13C	109.5
O8—C4—C3	109.6 (3)	H13B—C13—H13C	109.5
O8—C4—C5	107.7 (3)	O10—C14—C5	104.2 (3)
C3—C4—C5	108.8 (3)	O10—C14—H14A	110.9
O8—C4—H4	110.2	C5—C14—H14A	110.9
C3—C4—H4	110.2	O10—C14—H14B	110.9
C5—C4—H4	110.2	C5—C14—H14B	110.9
O1—C5—C14	106.8 (3)	H14A—C14—H14B	108.9
O1—C5—C4	109.7 (3)	O9—C15—O8	123.8 (5)
C14—C5—C4	113.9 (4)	O9—C15—C16	125.6 (5)
O1—C5—H5	108.8	O8—C15—C16	110.6 (4)
C14—C5—H5	108.8	C15—C16—H16A	109.5
C4—C5—H5	108.8	C15—C16—H16B	109.5
O3—C6—O2	122.7 (4)	H16A—C16—H16B	109.5
O3—C6—C7	124.6 (5)	C15—C16—H16C	109.5
O2—C6—C7	112.6 (5)	H16A—C16—H16C	109.5
C6—C7—C8	107.7 (5)	H16B—C16—H16C	109.5
C6—C7—H7A	110.2	O11—C17—O10	121.9 (5)
C8—C7—H7A	110.2	O11—C17—C18	126.5 (5)
C6—C7—H7B	110.2	O10—C17—C18	111.6 (4)
C8—C7—H7B	110.2	C17—C18—H18A	109.5
H7A—C7—H7B	108.5	C17—C18—H18B	109.5
C9—C8—C7	111.6 (7)	H18A—C18—H18B	109.5
C9—C8—H8A	109.3	C17—C18—H18C	109.5
C7—C8—H8A	109.3	H18A—C18—H18C	109.5
C9—C8—H8B	109.3	H18B—C18—H18C	109.5
C7—C8—H8B	109.3		
			
C6—O2—C1—O1	−99.1 (4)	C1—O1—C5—C14	−173.6 (3)
C6—O2—C1—C2	141.5 (4)	C1—O1—C5—C4	62.5 (4)
C5—O1—C1—O2	−178.6 (3)	O8—C4—C5—O1	59.6 (4)
C5—O1—C1—C2	−61.7 (4)	C3—C4—C5—O1	−59.2 (4)
C10—O4—C2—C3	−134.7 (4)	O8—C4—C5—C14	−60.1 (4)
C10—O4—C2—C1	107.5 (4)	C3—C4—C5—C14	−178.9 (3)
O2—C1—C2—O4	−69.9 (4)	C1—O2—C6—O3	4.1 (7)
O1—C1—C2—O4	174.5 (3)	C1—O2—C6—C7	−178.8 (4)
O2—C1—C2—C3	172.6 (3)	O3—C6—C7—C8	−96.6 (7)
O1—C1—C2—C3	57.0 (4)	O2—C6—C7—C8	86.3 (6)
C12—O6—C3—C2	−156.7 (3)	C6—C7—C8—C9	179.1 (7)
C12—O6—C3—C4	81.5 (4)	C2—O4—C10—O5	1.4 (7)
O4—C2—C3—O6	65.8 (4)	C2—O4—C10—C11	−179.6 (4)
C1—C2—C3—O6	−177.2 (3)	C3—O6—C12—O7	1.4 (6)
O4—C2—C3—C4	−171.4 (3)	C3—O6—C12—C13	−179.5 (5)
C1—C2—C3—C4	−54.4 (4)	C17—O10—C14—C5	−164.4 (4)
C15—O8—C4—C3	−104.4 (4)	O1—C5—C14—O10	177.8 (3)
C15—O8—C4—C5	137.4 (4)	C4—C5—C14—O10	−60.9 (5)
O6—C3—C4—O8	59.4 (4)	C4—O8—C15—O9	2.7 (7)
C2—C3—C4—O8	−61.5 (4)	C4—O8—C15—C16	−178.6 (4)
O6—C3—C4—C5	177.0 (3)	C14—O10—C17—O11	−0.2 (7)
C2—C3—C4—C5	56.1 (4)	C14—O10—C17—C18	179.0 (4)

### Hydrogen-bond geometry (Å, °)

**Table:**

*D*—H···*A*	*D*—H	H···*A*	*D*···*A*	*D*—H···*A*
C3—H3···O11^i^	0.98	2.47	3.362 (5)	152
C5—H5···O11^i^	0.98	2.57	3.443 (6)	149
C11—H11B···O5^ii^	0.96	2.49	3.293 (7)	141
C16—H16C···O9^iii^	0.96	2.60	3.441 (7)	147

Symmetry codes: (i) −*x*, *y*+1/2, −*z*; (ii) −*x*+1, *y*+1/2, −*z*+1; (iii) −*x*+1, *y*−1/2, −*z*.
